# The Combined Applications of Microbial Inoculants and Organic Fertilizer Improve Plant Growth under Unfavorable Soil Conditions

**DOI:** 10.3390/microorganisms11071721

**Published:** 2023-06-30

**Authors:** Malek Al Methyeb, Silke Ruppel, Bettina Eichler-Löbermann, Nikolay Vassilev

**Affiliations:** 1 Faculty of Agricultural and Environmental Sciences, University of Rostock, Justus-von-Liebig Weg 6, D-18051 Rostock, Germany; 2 Leibniz Institute of Vegetable and Ornamental Crops (IGZ), Theodor-Echtermeyer-Weg 1, D-14979 Grossbeeren, Germany; 3 Institute of Biotechnology, Department of Chemical Engineering, University of Granada, 18071 Granada, Spain

**Keywords:** microbial inoculants, arbuscular mycorrhizal fungi, *Kosakonia radicincitans*, organic fertilizer, field experiment, soil conditions, soil pH, yield

## Abstract

The performance of two bio-inoculants either in single or in combined applications with organic fertilizer was tested to determine their effect on plant growth and yield under normal and unfavorable field conditions such as low pH value and low content of P. Arbuscular Mycorrhiza Fungi (AMF; three species of *Glomus*) and the plant-growth-promoting bacterial strain *Kosakonia radicincitans DSM16656* were applied to barley in a two-year field experiment with different soil pH levels and available nutrients. Grain yield; contents of P, N, K, and Mg; and soil microbial parameters were measured. Grain yield and the content of nutrients were significantly increased by the applications of mineral fertilizer, organic fertilizer, AMF, and *K. radicincitans*, and the combined application of organic fertilizer with AMF and with *K. radicincitans* over the control under normal growth conditions. Under low-pH and low-P conditions, only the combined application of the organic fertilizer with *K. radicincitans* and organic fertilizer with AMF could increase the grain yield and content of nutrients of barley over the control.

## 1. Introduction

The application of microbial inoculants in agricultural systems is earning more interest in modern agriculture, since these inoculants have the potential to improve plant growth and enhance the availability of nutrients in soil [1,2]. It was reported that the application of bio-inoculants could improve plant growth even under unfavorable soil conditions such as acidic soils with a low content of available nutrients [3]. Acidic soil is considered to be a major problem in arable lands worldwide [4,5,6]. Soil acidity could have many negative effects such as reducing soil structure quality, decreasing the availability of essential nutrients such as phosphorus [7], increasing soil toxicity due to the release and accumulation of toxic elements such as aluminum [8], and inhibiting beneficial communities of the microorganisms in soil [9].

Although the plant beneficial role of fungi is, in general, understudied [10], it is well established that arbuscular mycorrhizal fungi (AMF), which form a beneficial symbiotic relationship with most crop plants [11,12], are able to improve the availability of soil nutrients [13], reduce nutrient leaching [14], improve soil structure [15], and improve plant tolerance to biotic and abiotic stress [13]. AMF can also increase plant growth and resistance under suboptimal acidic soil conditions [3].

Plant-growth-promoting rhizobacteria (PGPR) are a promising potential tool to sustainable agricultural production [16,17]. PGPR are a group of bacteria that can improve growth by different mechanisms [18,19]. Many species of PGPR enhance the availability of essential nutrients and improve the efficiency of the applied nutrients [20,21], provide growth hormones to the plants [22,23], and improve plant resistance against pathogens [24] and abiotic stress [25].

*Kosakonia radicincitans* (formerly *Enterobacter radicincitans*) is a bacterium belonging to the PGPR, which is able to colonize plant surfaces and tissues [26]. *K. radicincitans* can provide many advantages to plants due to its ability to fix atmospheric N, solubilizing P, producing growth hormones, and inhibiting pathogenic fungi [27,28,29,30].

Activities and advantages provided by microorganisms in the soil are highly correlated with the soil conditions such as soil content of the available nutrients and the organic matter (OM). It was reported that the application of organic fertilizer can improve the microbial activity in soil [31] and the soil structure [32], and increase the soil pH value after application to acidic soil [33].

However, the effect of the application of microbial inoculants in combination with organic fertilizer could be different depending on the soil pH and content of available nutrients. The aim of this work is to investigate the differentiation of barley plant responses to the application of microbial inoculants containing AMF and/or PGPR alone or in combination with cattle manure in soils with intermediate and low pH values.

## 2. Materials and Methods

### 2.1. Site Description

A field experiment was established in Rostock, northern Germany, about 15 km in-land from the Baltic Sea (54°3′41.47″ N; 12°5′5.59″ E). The study area is strongly affected by marine conditions. The total annual precipitation was 73.9 cm in 2017 and 46.6 cm in 2018. The mean annual temperature was 10.2 °C in 2017 and 10.8 °C in 2018. The soil texture is loamy sand and the dominating soil type on the site is a stagnic cambisol. Two sites with different soil properties were selected with the main characteristics of both sites presented in Table 1. The soil material was sampled from a depth of 0–20 cm.

### 2.2. Microbial Inoculants

#### 2.2.1. Arbuscular Mycorrhizal Fungi

The AMF preparation used was a commercial product (Mycorrhiza granulates from the company INOQ GmbH in Germany). The AMF product was a mix of three *Glomus* species (*Glomus etunicatum*, *G. intraradices*, and *G. claroideum*) with a spore concentration of 10^5^ L^−1^. The carrier material was expanded clay with a grain size of 2 to 4 mm and pH value of 7.5. According to the manufacturer’s instructions, 100 mL of the AMF preparation was applied per square meter of soil.

The plant inoculation process with AMF was different between the two experiments.

In the first experiment, the barley seeds were treated with the fungicide Aagrano (chemical compound Imazalil), and AMF spores were added into the root zones of the young plants four weeks after sowing to avoid the negative effect of the fungicide on AMF growth. Cracks in the soil among the plant rows were made manually using a mattock and then the AMF spores were added into the soil along the rows. Following this, the soil was introduced back over the spores.

In the second experiment, the seeds were not treated with fungicide, to avoid the delay of AMF addition. Instead, barley seeds were treated using X-rays in the Fraunhofer Institute for Electron Beam and Plasma Technology in Dresden, Germany. Using this technology, seeds are treated with low-energy electrons for seed dressing to inactivate the pathogenic organisms on their surfaces and in the seed coats [34]. Therefore, the application of AMF was possible without delay. AMF were added into the seeding depth using the sowing machine directly before the seeds.

#### 2.2.2. *Kosakonia radicincitans*

The bacterial inoculant was prepared at the microbiology laboratory of the Leibniz Institute of Vegetable and Ornamental Crops Groβbeeren (IGZ), Germany. *K. radicincitans* cells were grown in a standard nutrient solution (MERCK 1) at 29 °C in a rotary incubator at 100 rpm for 48 h [30].

The seeds were soaked in a bacterial suspension (10^8^ cells mL^−1^) for 5–10 min. Afterward, they were dried in the dark at room temperature. During the two-leaf growth stage of the plants, the bacterial suspension (10^8^ cells mL^−1^) was additionally sprayed with a hand pump onto the young plants (1 mL per plant) in all experiments. The aim of the second inoculation was to improve the opportunity for the bacterial cells to colonize and establish on the plants, as well as successfully compete with the native bacterial communities.

### 2.3. Experimental Design

Plots were prepared and distributed randomly in four replicates. The single plot size was 12 m^2^ in 2017 (1.5 × 8 m) and 7.5 m^2^ (1.5 × 5 m) in 2018. Barley (*Hordeum vulgare*, Barke cultivar) was sown in mid-April 2017 and at the end of March 2018 at a density of 300 seeds m^−2^, as recommended for spring barley in Germany. Crop protection and weed control were not carried out (except the fungicide Aagrano), to avoid the effect of the respective chemical substances on the applied microorganisms. Seven treatments were applied as follows: (1) Ctrl (control, without any additions), (2) MF (mineral fertilizer; 120 kg ha^−1^ of calcium ammonium nitrate and 27% N, was added in two batches; the first application was 80 kg ha^−1^ added directly after sowing, and 40 kg ha^−1^ was added five weeks after the first application), (3) KR (*K. radicincitans*), (4) AMF, (5) OF (organic fertilizer; 3 L of cattle manure in liquid form was applied per square meter), (6) OF + KR, and (7) OF + AMF. MF treatment was experimented to compare its effect on barley with the OF treatment and was not applied afterward alone or in combination with microorganisms. Characteristics and nutrient content of the organic fertilizer are presented in Table 2. The manure was analyzed at LUFA laboratory (Agricultural Analysis and Research Institute in Mecklenburg—Vorpommern).

### 2.4. Plant and Soil Analysis

Three random soil samples were collected from each plot before sowing. The soil samples were dried at room temperature and then sieved using a 2 mm sieve.

For pH determination, 10 g of the sieved soil was mixed with 25 mL of 0.01 N CaCl_2_ in a flask, then the suspension was stirred with a glass rod, and after 30 min, the suspension was stirred again and then filtered. pH value was measured after 1 h, using an electrode (pH Electrode SenTix 81, Sensor technique Meisberg GmbH).

Soil organic matter was determined by drying fine soil at 105 °C for 4 h, and then the samples were weighed (w1). Afterward, the samples were put into a muffle furnace at 550 °C for 4 h and weighed again (w2).

Soil organic matter (SOM) was calculated according to the equation:

SOM % = (w1 − w2)/w2 × 100

For P, K, and Mg determination, 10 g of air-dried soil was mixed in 125 mL of Doppel-Lactate (DL), and the solution was shaken for 1.5 h and then filtered. Then, 25 mL of the filtrated soil solution was mixed with 15 mL vanadate-molybdate mixture and 50 mL of DL solution in a volumetric flask. After 2 h, P was measured by spectrophotometer at a wavelength of 430 nm (Spekol 11, Carl Zeiss, Jena, Germany). Soil-filtrated suspension was also used for K and Mg determination: K was measured using flame photometer (Elex 6361, Eppendorf, Hamburg, Germany) and Mg was measured using spectrometer (Epos Analyzer 5060, Com Eppendorf).

Barley plants were harvested 17 weeks after sowing in the first experiment in 2017 and after 16 weeks in the second experiment in 2018.

After harvest, plant seeds were dried for 96 h at 60 °C; following this, the sample of seeds was milled and prepared for chemical analyses. 2 g of the dry milled seeds were placed in a muffle furnace at 550 °C for 4 h; then, the ash was digested in 22 mL of HCl (25%) in 50 mL volumetric flasks and put on an electric heater for 15–20 min. After cooling, the digestion solution was supplemented with distilled water. Later, the solution was filtered into 50 mL flasks. After filtration, 10 mL from the solution was put into 100 mL volumetric flasks and then the flasks were filled up with distilled water. Afterward, 15 mL from the solution was transferred into 50 mL volumetric flasks and the volume was supplemented with vanadate-molybdate mixture. After 2 h, P was measured using spectrophotometer at a wavelength of 430 nm (Spekol 11, Carl Zeiss, Jena, Germany). K and Mg were estimated from the filtered suspension using flame photometer (Elex 6361, Eppendorf) for K and spectrophotometer (Epos Analyzer 5060, Com Eppendorf) for Mg. Nitrogen was analyzed as total N using modified Kjeldahl digestion method.

#### 2.4.1. Soil Sampling and Microbial Analyses

After harvest, three random soil samples were collected from each plot (0–30 cm soil layer). Soil samples were sieved to 2 mm and stored at −20 °C until the microbial parameters were measured.

#### 2.4.2. Soil Microbial Measurements

Substrate-induced respiration (SIR) method was applied to measure the soil microbial biomass and the basal respiration. An infrared gas analyzer was used for the measurements [35]. The operating principle of the infrared gas analyzer offers an automated system for continuous soil respiration and microbial biomass measurements based on infrared gas analysis. The switching device is controlled by a computer and allows for taking measurements each hour of up to 24 samples when switching intervals of 2.5 min are selected. This allows the use of the SIR method for biomass determination. The system was run by using software [35].

The soil microbial biomass carbon (C_mic_) content of soil samples (100 g of wet soil, 50% water holding capacity) was calculated according to the correlation of SIR with the fumigation incubation method. The soil was mixed with glucose (2 mg g^−1^ soil) and analyzed under a continuous gas flow at 20 °C ± 1 K. C_mic_, which includes all respiratory active soil organisms that are able to metabolize glucose and is expressed as μg C_mic_ g^−1^ dry soil.

Soil basal respiration was measured using the infrared gas analyzer without the addition of substrates (20 °C ± 1 K) and expressed as μg CO_2_-C g^−1^ dry soil h^−1^.

#### 2.4.3. P-Solubilizing Bacteria

The most probable number of P-solubilizing bacteria was determined using dilution and plating method [36]. The isolated microorganisms were tenfold-diluted 5 times in sterile 0.05 M NaCl. Amounts of 100 μL of succeeding dilutions were streaked onto solid Muroveć nutrient medium in three replicates and incubated at 29 °C for two weeks [37]. Muroveć medium consists of (g L^−1^) K_2_SO_4_ 0.2, MgSO_4_* 7H_2_O 0.4, agar-agar 20, glucose 10, and L-asparagine 1 (both separately filter-sterilized and added after autoclaving and cooling down the medium to 60 °C); simultaneously, CaCl_2_ 2.2 and Na_3_PO_4_ × 12H_2_O 3.8 were mixed by consistently shaking the medium to precipitate calcium phosphate. After one and two weeks, colonies inducing pellucid zones in the medium (zones of P-solubilizing activity) were counted. The number of P-solubilizing bacteria was calculated per g dry soil according to the MPN method.

### 2.5. Statistical Analysis

All statistical analyses were carried out with four replications and the mean values of the four replicates. Soil sampling was carried out with four replications and with the mixture of three samples of each replicate. The data in all the experiments were subjected to a one-way analysis of variance. One-way ANOVA was performed to test the differences among the treatments. The mean values were compared with a post hoc test followed by Tukey‘s HSD test at *p* < 0.05. The data were analyzed using Statistica 6.0 (StatSoft 2001) software.

## 3. Results

### 3.1. Crop yield and Nutrient Uptake

**Figure 1:**
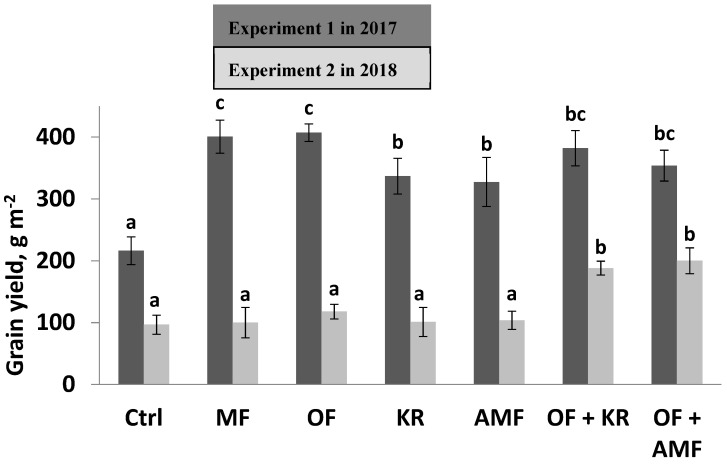
The effect of the single and the combined applications on barely grain yield (g m^−2^) in the first field experiment (experiment 1) in 2017 and the second field experiment (experiment 2) in 2018; Ctrl (control without any application), mineral fertilization (MF), organic fertilization (OF), arbuscular mycorrhiza fungi (AMF), and *K. radicincitans* (KR); bar graphs with different letters are significantly different according to Tukey’s test at *p* ≤ 0.05.

In the first experiment when the acidity of soil was medium, the application of single microbial inoculants without other additives enhanced the growth and grain yield of barley. *K. radicincitans* application increased the yield up to 56% over the control, whereas the yield was increased by 51% over the control by AMF inoculation (Figure 1). Grain yield was significantly increased in the treatments of the mineral fertilizer or the organic fertilizer compared to the control treatment in the first experiment (Figure 1). The single application of the cattle manure enhanced the grain yield of barley up to 88% over the non-fertilized control. Similarly, the mineral fertilizer application increased barley yield (85%) compared to the control. The uptake of the nutrients in seeds was significantly affected by the application of the microbial inoculants (Table 3). The content of P and N was significantly increased after the application of AMF or the *K. radicincitans* over the control.

The single application of either mineral or organic fertilizers improved nutrients (P, N, K, and Mg) uptake in comparison to the control in the first experiment (Table 3). The combined application of the organic fertilizer with AMF or with *K. radicincitans* had no significant effect either on grain yield or on nutrient uptake in comparison to the single application of the organic fertilizer, AMF, the *K. radicincitans*, or the control in the first experiment (Figure 1, Table 3).

In contrast to the first experiment, the single application of either mineral fertilizer or the manure had no effect on grain yield or nutrient uptake in the second experiment when the soil pH value was 4.9 and the P content was low (Figure 1 and Table 4). Grain yield was significantly higher by the combined application of the organic fertilizer with AMF or *K. radicincitans* compared to the single applications of mineral fertilizer, organic fertilizer, AMF, *K. radicincitans*, and the control (Figure 1).

Barley grain yield was significantly enhanced by the combined application of the cattle manure with the microbial inoculants (Figure 1). The combined application of the organic fertilizer and *K. radicincitans* increased the grain yield 95% over the control, 60% over the single application of the organic fertilizer, and 86% over the single application of the *K. radicincitan*. The combined treatment of the organic fertilizer and AMF improved the grain yield by 106% over the non-treated control, 86% over the single application of the manure, and 93% over the single application of AMF (Figure 1). The combined application of the organic fertilizer with AMF or with *K. radicincitans* affected positively nutrient uptake (Table 4). P, N, K, and Mg increased significantly by the application of the organic fertilizer with *K. radicincitans* in comparison to the control or the single application of *K. radicincitans*. N and Mg uptake was significantly higher over the single application of the organic fertilizer or the *K. radicincitans*. Combined application of the organic fertilizer with AMF enhanced significantly N and Mg uptake in comparison to the control or the single application of the AMF (Table 4).

#### Effect on Soil Microbial Parameters

The values of soil microbial parameters were much higher in the first experiment than in the second experiment (Table 5 and Table 6). However, the experimental treatments did not significantly affect the microbial basal respiration activity nor the soil microbial biomass content (Table 5 and Table 6).

Only under low-soil-pH conditions did the application of cattle manure alone and in combination with AMF and *K. radicincitans* induce higher microbial activities and soil microbial biomass, which was not significant, due to the high variability under field experimental conditions (Table 6). The number of P-solubilizing bacteria significantly increased after the combined application of the cattle manure and *K. radicincitans* in the first experiment in comparison to the single applications of the organic manure or the non-inoculated control (Table 5). In the second experiment, the metabolic quotient was higher after the application of AMF singularly or in combination with the organic fertilizer in comparison to the control or the other treatments, and the lowest value was registered after the combined application of the organic fertilizer and the *K. radicincitans* (Table 6).

## 4. Discussion

In general, the presence and type of substrates and formulation additives are important factors for the growth, establishment, and activity of plant beneficial microorganisms [2,38]. Plant growth and yield usually have many advantages by the application of the mineral fertilizer, organic fertilizers, and bio-inoculants, or by the combined application of the organic fertilizers and the bio-inoculants. Our results proved that the combined application of the organic fertilizer with the bio-inoculants, either with AMF or with *K. radicincitans*, significantly improved grain yield and nutrient uptake under both soil pH and P content conditions. On the other hand, the single treatments of mineral fertilizer, organic fertilizer, AMF, and *K. radicincitans* contributed significantly to grain yield and nutrient uptake at the first experiment when the soil pH value was medium and the P content was higher (except the content of K and Mg with AMF and *K. radicincitans*). As MF treatment demonstrated the same effectivity as the OF in both experiments (2017, 2018), alone or combined with the biofertilizers, and bearing in mind the high price of all chemical fertilizers (particularly due to the war in Ukraine), treatments with MF combined with the AM and bacterial products were not carried out.

The effect of single applications of the mineral fertilizer, organic fertilizer, AMF, and *K. radicincitans* on the growth and yield of barley at unfavorable soil conditions (low pH and content of nutrients) was insignificant. The AM effect should be analyzed as, independent of the high inoculum amount applied in both experiments, the measured parameters were different. The observed differences in plant response in AM treatment may result from variations in environmental conditions. In general, plant growth and soil microbial activity are dependent on pH. Mycorrhizal fungi have an extensive extra-radical mycelial network in the soil. However, soil pH produces a strong selective pressure structuring an AM fungal niche space on mycelia [39], forming more extraradical mycelium at the higher pH but almost no detectable extraradical mycelium at lower pH. In another, more recent study, the close relationship between the functionality of AMF and arbuscule abundance was demonstrated, with the latter being greatly reduced in soil with low pH [40].

On the other hand, soil pH is known to have considerable influence on plant growth because it affects the mobilization and availability of nutrients. Therefore, the low soil pH value and low content of nutrients in the second experiment (2018) could explain the low values of studied plant parameters. It was repeatedly reported that soil pH values below 5.0 are considered as a plant growth impediment [40], since plant growth will be disturbed due to negative effects related to low soil pH such as deficiencies of essential nutrients and mineral toxicity [41,42]. In our study, the application of organic manure resulted in an increase in both soil pH value [43,44] and organic matter content [45], thus improving the conditions (higher pH value and organic matter as an energy source for the microbes) for bio-inoculant development. These conditions could explain why the combined application of the manure and AMF or *K. radicincitans* in the second experiment increased plant growth more than the single applications. Barley plants showed a positive response to the single inoculation of either AMF or *K. radicincitans* when soil was medium-acidic and had a sufficient content of available P in the first experiment. The application of the bio-inoculants to an arable soil with sufficient content P could improve plant growth under conventional agricultural systems, since these soils probably have a poor AMF community [46] and hence the applied AMF inoculum could be able to improve the diversity of the AMF in soil and to establish symbiotic interactions with plant roots [47].

Soil pH is a very important factor that could affect microbial activity [48]. The application of the microbial inoculants affected the soil microbial parameters in both experiments, but in general these parameters were more pronounced in the first experiment compared to the second experiment. This difference could be due to the fact that soil conditions (mainly soil pH) were more suitable for microbial growth and activity in the first experiment than in the second experiment.

## 5. Conclusions

As a conclusion, this study demonstrated that the effect of bio-fertilizers and organic fertilizer is dependent on the soil conditions. The combined application of the bio-inoculants with organic fertilizer helped plants to grow under conditions of acidic soils. The single application of the mineral fertilizer, the organic fertilizer, AMF, and *K. radicincitans* had no significant effect when the soil pH and the content of nutrients were low, while the combination of the organic fertilizer either with AMF or with *K. radicincitans* could significantly improve the yield and content of nutrients. The effect of the same applications was different under moderate soil pH since the single application of the mineral fertilizer, organic fertilizer, or the bio-inoculants increased the yield and content of nutrients, but the combined application of the bio-inoculants with the organic fertilizer did not further synergistically enhance this effect.

The preliminary results of this study gave the first indication of possible soil quality impacts on microbial plant interactions, which however should be proven with formulated microorganisms.

## Figures and Tables

**Table 1:** Main properties of soil at the experimental sites of the field experiments in 2017 and 2018.

	pH	SOM	P	K	Mg
Site 1 (Experiment 2017)	5.8	2.27	6.27	7.40	14.10
Site 2 (Experiment 2018)	4.9	2.23	2.87	4.51	23.26

P, K, and Mg in mg 100 g^−1^ soil; SOM: soil organic matter (%).

**Table 2:** Characteristics and nutrient contents of the cattle manure in g L^−1^.

Parameter	Experiment 1	Experiment 2
Dry substance	54.11	86.00
pH (value)	7.90	7.90
N	2.20	3.40
P (as P_2_O_5_)	1.50	2.02
K (as K_2_O)	2.90	4.16
Mg (as MgO)	1.03	1.03

N, P, K, and Mg estimated in g L^−1^. Experiment 1 in year 2017. Experiment 2 in year 2018.

**Table 3:** Uptake of N, P, K, and Mg in grain (g m^−2^) in the different treatments in barley field experiment in 2017.

Treatment	P	N	K	Mg
Ctrl	0.89 a	3.27 a	0.87 a	0.28 a
MF	1.83 d	8.17 d	1.78 b	0.56 bc
OF	1.76 cd	7.20 cd	2.02 b	0.61 c
KR	1.37 b	5.50 b	1.33 ab	0.42 ab
AMF	1.40 bc	5.49 b	1.37 ab	0.43 abc
OF + KR	1.60 bcd	6.12 bc	1.62 b	0.52 bc
OF + AMF	1.50 bcd	5.88 bc	1.51 ab	0.46 bc

Note: Ctrl = control, KR = *K. radicincitans*, AMF = arbuscular mycorrhiza fungi, OF = organic fertilizer applied, MF = mineral nutrients applied. Reported data are the mean of 4 replications, and values in each column with different letters are significantly different according to Tukey’s test at *p* ≤ 0.05.

**Table 4:** Grain content of N, P, K, and Mg (g m^−2^) in the different treatments in barley field experiment in 2018.

Treatment	P	N	K	Mg
Ctrl	0.41 a	1.80 a	0.35 a	0.14 a
MF	0.42 a	2.25 ab	0.37 a	0.14 a
OF	0.48 ab	2.30 ab	0.44 ab	0.17 ab
KR	0.42 a	1.93 a	0.36 a	0.13 a
AMF	0.38 a	1.99 a	0.35 a	0.13 a
OF + KR	0.75 b	3.57 c	0.65 b	0.26 c
OF + AMF	0.68 ab	3.32 bc	0.59 ab	0.24 bc

Note: Ctrl = control, KR = *K. radicincitans*, AMF = arbuscular mycorrhiza fungi, OF = organic fertilizer, MF = mineral nutrients. Reported data are the mean of 4 replications and values in each column with different letters are significantly different according to Tukey’s test at *p* ≤ 0.05.

**Table 5:** Soil microbial parameters in the different treatments in barley field experiment in 2017.

Treatment	BR	SMB	MQ	PSB
Ctrl	6.36 a	130.8 a	48	1.39 × 10^7^ a
OF	7.23 a	135.1 a	53	1.47 × 10^7^ a
KR	8.30 a	122.1 a	68	1.63 × 10^7^ ab
AMF	8.22 a	136.0 a	60	1.42 × 10^7^ a
OF + KR	7.40 a	160.1 a	46	2.85 × 10^7^ b
OF + AMF	7.21 a	156.8 a	46	1.48 × 10^7^ a

Note: Ctrl = control, KR = *K. radicincitans*, AMF = arbuscular mycorrhiza fungi, OF = organic fertilizer applied, BR: basal respiration (µg CO_2_-C (g^−1^ soil h^−1^)); SMB: soil microbial biomass µg C g^−1^ soil; MQ: metabolic quotient μg CO_2_-C/mg C_mic_h^−1^; PSB = phosphate-solubilizing bacteria (bacterial cells g^−1^ soil). Reported data are the mean of 4 replications and values in each column with different letters are significantly different according to Tukey’s test at *p* ≤ 0.05.

**Table 6:** Soil microbial parameters in the different treatments in barley field experiment in 2018.

Treatment	BR	SMB	MQ	PSB
Ctrl	4.22 a	70.5 a	60	3.08 × 10^5^ a
OF	5.11 a	93.9 a	54	4.92 × 10^5^ a
KR	4.64 a	77.2 a	60	2.33 × 10^6^ a
AMF	4.56 a	70.1 a	65	1.18 × 10^6^ a
OF + KR	5.24 a	101.5 a	51	1.68 × 10^6^ a
OF + AMF	5.98 a	91.7 a	65	n.d.

Note: Ctrl = control, KR = *K. radicincitans*, AMF = arbuscular mycorrhiza fungi, OF = organic fertilizer applied, n.d. = not determined, BR: basal respiration (µg CO_2_-C (g^−1^ soil h^−1^)); SMB: soil microbial biomass µg C g^−1^ soil; MQ: metabolic quotient μg CO_2_-C/mg C_mic_h^−1^; PSB = phosphate-solubilizing bacteria (bacterial cells g^−1^ soil). Reported data are the mean of 4 replications, and values in each column with different letters are significantly different according to Tukey’s test at *p* ≤ 0.05.

## Data Availability

Not applicable.
